# Attachment insecurity, heart rate variability, and perceived social support in a diverse sample of young adults

**DOI:** 10.3389/fpsyg.2023.1208924

**Published:** 2023-11-10

**Authors:** Vida Pourmand, Nicole M. Froidevaux, DeWayne P. Williams, Ilona S. Yim, Belinda Campos

**Affiliations:** ^1^Department of Psychological Science, University of California, Irvine, Irvine, CA, United States; ^2^Department of Chicano/Latino Studies, University of California, Irvine, Irvine, CA, United States

**Keywords:** attachment, heart rate variability, social support, culture, ethnicity, Latino

## Abstract

Psychological and physical factors are robustly associated with perceived social support. Drawing from the literature on attachment style in adults and psychophysiology, we examined the possibility that the interaction of attachment insecurity and resting heart rate variability (HRV) was associated with perceived social support in a diverse sample of young adults living in the U.S (*N* = 145, *M*_age_ = 20.45) that was majority Latino (*n* = 77). Analyses revealed three key findings. First, in the overall sample, attachment avoidance and attachment anxiety were negatively associated with perceived social support, but in the Latino sample, only attachment avoidance was negatively associated with perceived social support. Second, HRV was not associated with perceived social support in the overall sample nor in the Latino sample. Third, attachment insecurity and HRV interacted to predict perceived social support only in the Latino sample such that, for those with lower levels of HRV, attachment anxiety was positively associated with perceived social support. This study underscores the importance of examining both psychological and physiological processes with careful consideration of ethnicity/culture in order to better understand perceived social support.

## 1. Introduction

Perceived social support, the perception that one is cared for and has access to a reliable social network, is robustly related to mental and physical well-being. Higher perceived social support is associated with relational benefits such as higher relationship satisfaction (Collins and Feeney, 2000, 2004) as well as beneficial health outcomes such as decreased risk for early mortality (Shor et al., 2013; Freeborne et al., 2019). Despite these benefits, less is known about psychological and physiological processes that shape perceived social support. This is important, as *perceived* social support is associated with favorable outcomes, including mortality and morbidity, more consistently than *received* social support (Uchino, 2009). Two self-regulatory processes, attachment insecurity and resting heart rate variability (HRV), may be important for understanding influences on perceived social support. The goal of the current study was to examine how attachment insecurity and resting HRV *together* was associated with perceived social support in a diverse young adult sample that contained a sizeable majority of Latino participants. The diversity of the sample also allowed us to explore the possibility of ethnic/cultural variation in these links, a focus that is important for arriving at a more nuanced understanding of perceived social support.

### 1.1. Predictors of perceived social support

#### 1.1.1. Attachment insecurity

Attachment is developed in infancy as a means of survival; infants rely on their primary caregiver for shelter and sustenance as well as comfort during sickness and safety from threat (Bowlby, 1979; Mikulincer and Shaver, 2010). Over the course of the early years of life, infants develop internal working models of what to expect from caregivers during times of distress and these models vary depending on the type of caregiving infants receive (Bowlby, 1976; Mikulincer and Shaver, 2010). These internal working models become the lens through which adults perceive the social world around them (see Pietromonaco and Barrett, 2000 for a review). When provided with sensitive and responsive care, infants develop a healthy or secure attachment to their caregiver which allows them to perceive the world, and future romantic partners, as reliable and worthy of trust (e.g., Hazan and Shaver, 1987; Gillath and Karantzas, 2019). In the absence of this type of care, attachment insecurity can develop.

In the literature on attachment style in adults, attachment insecurity is divided into two continuous facets, attachment avoidance and attachment anxiety. Higher levels of either of these facets represents higher insecurity with lower levels representing higher attachment security. When a caregiver responds to a child’s needs with minimal, nonexistent, or dismissive care, children develop higher levels of attachment avoidance (Ainsworth, 1978; Shemmings, 2006). In adults, attachment avoidance presents with a preference for isolation and distance in relationships as well as a desire to avoid dependence (Brennan et al., 1998; Moore and Leung, 2002; Crowell et al., 2008). When a caregiver responds to a child’s needs with inconsistent or insensitive care, children develop higher levels of attachment anxiety (Ainsworth, 1978; Shemmings, 2006). In adults, attachment anxiety presents with a higher negative self-image, an uncontrollable deep concern of abandonment, and a persistent need for affirmation from others (Moore and Leung, 2002; Crowell et al., 2008). As such, attachment insecurity is robustly associated with perceived social support (Anders and Tucker, 2000; Collins and Feeney, 2004; Jakubiak et al., 2023). For example, higher attachment avoidance is associated with lower perceived social support, and this pattern is similar yet slightly weaker for the association of attachment anxiety with perceived social support (Mallinckrodt and Wei, 2005; Mikulincer and Shaver, 2009; Stanton and Campbell, 2014). Attachment styles and self-regulation are well known to covary, a link that highlights attachment styles’ role as an important aspect of self-regulation (e.g., Orehek et al., 2017; Pallini et al., 2018).

#### 1.1.2. Heart rate variability

Physiological processes that are relevant to self-regulation are also implicated in perceived social support. Indeed, the neural underpinnings of these associations are well documented. For example, stronger connectivity between cortical brain regions such as the prefrontal cortex, and subcortical brain regions such as the amygdala, signal better self-regulation (Etkin et al., 2015), and is associated with higher perceived social support (Che et al., 2014; Sato et al., 2020). These connections highlight that brain regions modulate the autonomic nervous system (Thayer and Lane, 2000; Appelhans and Luecken, 2006; Thayer et al., 2009) and, as described in the paragraph below, the autonomic nervous system has been associated with perceived social support (e.g., Goodyke et al., 2022).

The aforementioned brain regions involved in self-regulatory processes are structurally and functionally associated with the parasympathetic, or rest-and-digest, branch of the autonomic nervous system (see Thayer et al., 2012 for a review). Resting HRV^1^ is a non-invasive index of parasympathetic activity, and serves as a reliable psychophysiological marker of prefrontal activity and self-regulatory abilities (Thayer and Lane, 2000; Appelhans and Luecken, 2006; Thayer et al., 2009, 2012). Lower HRV is considered to be less adaptive, while higher HRV is considered more adaptive, both physiologically and psychologically (Appelhans and Luecken, 2006; Thayer et al., 2009, 2012; Williams et al., 2015; Visted et al., 2017). Empirical evidence to date indicates a positive association of HRV with perceived social support (Gerteis and Schwerdtfeger, 2016; Goodyke et al., 2022; Kvadsheim et al., 2022). This work suggests that better self-regulatory abilities and thus better socio-emotional regulation, as indexed by higher HRV, are associated with more favorable interpersonal relationships (i.e., higher perceived support).

### 1.2. Interplay of attachment insecurity and resting HRV in shaping social support

The association of higher attachment insecurity with lower perceived social support appears to be consistent in the literature (Mallinckrodt and Wei, 2005; Mikulincer and Shaver, 2009). Studies also suggest that higher HRV under resting and other conditions (i.e., reactivity) associated with higher perceived social support (e.g., Goodyke et al., 2022). Yet, the few empirical studies on the direct association of attachment insecurity with HRV are mixed (e.g., Maunder et al., 2006). At this time, the extent to which attachment insecurity and resting HRV operate *together* (i.e., interact) to shape perceived social support is unknown but the evidence suggests that this possibility is worthy of further examination.

Importantly, resting HRV is conceptualized as an endophenotype (Beauchaine and Thayer, 2015), such that it serves as an individual difference measure of how individuals both interpret and react to various psychosocial scenarios. In other words, resting HRV is considered a biomarker that assesses individual differences in the ability to engage in self-regulation processes (e.g., Thayer et al., 2009), which can alter how psychological and social factors may relate to one another. This claim is not without empirical evidence, as others have suggested that the way in which someone self-regulates in the moment, largely depends on their resting HRV (for discussion, see Park et al., 2013; Williams et al., 2016). Recent literature supports this thinking, as emerging theory has guided researchers to examine the role of HRV as a moderator of psychological and social factors (e.g., Kocsel et al., 2022). For example, one study examined how HRV moderated the link between discrimination and Canadian acculturation (Doucerain et al., 2022). Another study examined how HRV moderated the association of attachment insecurity with self-concept recovery (Sbarra and Borelli, 2013). Although the associations studied in the recent literature thus far vary from that of the current study, they lend evidence to the idea that HRV is relevant to the association of attachment insecurity with perceived social support.

### 1.3. Ethnicity/culture

One key source of variation to consider in perceived social support is cultural context (e.g., Kim et al., 2008). Cultural context shapes psychological processes and interpersonal relationships (Campos and Kim, 2017), and may play a role in the possible interaction of attachment insecurity and resting HRV with perceived social support. Moreover, Latinos are an ethnic group in whom patterns of attachment insecurity, HRV, and perceived social support may be distinct. Latino culture tends to value interconnectedness, prioritizing relationships, especially family relationships, over the personal desires of the self, and Latinos generally perceive high availability to socially supportive networks (Campos and Kim, 2017). At least one study has found that among U.S. Latinos, attachment avoidance is less endorsed and may be culturally incongruent (Campos et al., 2016). In contrast, attachment anxiety, which presents as a higher dependence on others and greater need for proximity and connection, may be less incongruent with Latino cultural norms; in this context, attachment anxiety may present fewer challenges to a person with this style of attachment insecurity. However, to our knowledge, this possibility has not been examined. Therefore, we explored the interaction of attachment insecurity and HRV on perceived social support in our sample of Latinos.

### 1.4. The current study

The current study examined the association of two self-regulatory processes, attachment insecurity (avoidance and anxiety) and resting HRV – both individually and in tandem – with perceived social support. We also explored these associations in a Latino sample specifically, the ethnic group that comprised our sample majority and for whom there is reason to expect distinctive patterns in the correlates of attachment insecurity. Our hypotheses were as follows:

1. First, that higher (H1a) attachment avoidance and (H1b) attachment anxiety would be associated with lower perceived social support in the overall sample.
2. Second, that higher (H2) HRV would be associated with higher perceived social support in the overall sample.
3. Third, that higher (H3a) attachment avoidance and (H3b) attachment anxiety would be associated with lower perceived social support in the overall sample, but that these associations would be moderated by HRV such that they would be weaker for those with higher levels of HRV.
4. Fourth, we explored the above associations in the Latino sample.

All hypotheses were pre-registered on the Open Science Framework (https://osf.io/j3dz9).

## 2. Method

### 2.1. Participants

One-hundred fifty-eight participants took part in a study of social responses to stress. Study participants were recruited via the social science subject pool at a university on the West Coast of the United States and from surrounding community colleges. Eligibility criteria for the study were that participants did not have any major medical condition (e.g., hypertension), did not abuse alcohol or tobacco (>5 cigarettes per day), did not have a math or speech phobia, and/or did not use any medication that could influence hypothalamic–pituitary–adrenal axis function. These exclusion criteria are based on the study’s research questions that focused on hypothalamic–pituitary–adrenal axis functioning (for more information on the study see, e.g., Campos et al., 2014; Busse et al., 2017; Yim et al., 2019).

The final sample included participants who had data on all key study variables (*N* = 145). Participants in the overall sample identified as male (*n* = 61) or female (*n* = 84), and the sample average age was 20.45 (SD = 1.98) years old. Participants self-identified as Black/African American (0.7%), Chinese Mainland (6.9%), Chinese Taiwan (1.4%), Indian (2.1%), Japanese (0.7%), Korean (6.2%), Mexican/Mexican-American/Chicano (42.1%), Other Latino (e.g., Guatemalan, Colombian; 11.0%), Pacific Islander (4.1%), Southeast Asian (4.8%), and White/Caucasian/European (14.5%). Participants who identified with multiple ethnicities were classified as being Multi-Ethnic (5.5%). Those who reported Mexican/Mexican-American/Chicano and Other Latino backgrounds were classified as Latino. The Latino sample (*n* = 77) consisted of 39 males and 38 females of an average age of 20.30 (SD = 2.01) years old.

### 2.2. Procedure

Data collection for this study was approved by the local Institutional Review Board. Participants were asked to not drink caffeine, eat, or participate in physically strenuous activity at least 1 h before their session. After providing written informed consent, six electrocardiogram (ECG) sensors were placed on participants’ torsos in a Lead-II configuration. To obtain resting HRV, participants engaged in a three-minute baseline period in which they sat quietly and breathed normally. Then, participants engaged in a laboratory stressor. After the stressor, participants completed self-report questionnaires. At the end of the session, participants were thanked, debriefed, and compensated.

### 2.3. Measures

#### 2.3.1. Attachment insecurity

A modified version of the Experiences in Close Relationships Revised (ECR-R; Fraley et al., 2000) scale was used to measure attachment insecurity. Nine items from each subscale were used (18-items out of the 36 original sale items). Participants reported on a seven-point Likert-type scale ranging from 1 (*strongly disagree*) to 7 (*strongly agree*). Three items were reverse scored to calculate attachment avoidance and one item was reverse scored to calculate attachment anxiety. All items from each subscale were averaged together to calculate composite scores, with higher scores indicating higher attachment avoidance (α = 0.85) and attachment anxiety (α = 0.90), respectively. This scale has shown to be a reliable scale in various populations, including among Latinos (Wei et al., 2004).

#### 2.3.2. Resting heart rate variability

ECG data was collected at a 1,000 Hz sampling rate using Biolab 2.4 and then was transferred into the Mindware HRV 3.0.25 software, where researchers edited ECG artifacts to prevent possible inaccuracies of peak detection automatically generated from the software. Following data cleaning procedures recommended by MindWare (MindWare Technologies), raw data was verified in 60-s segments, for the total length of the three-minute baseline. Once the ECG data were verified and double checked, high-frequency HRV (HF-HRV; 0.15–0.4 Hz) and the root mean square of successive differences between adjacent normal R-R intervals (RMSSD) were derived from the MindWare software. High-frequency HRV is a valid frequency-domain measure and RMSSD is a valid time-domain measure of parasympathetic influence on the heart (Malik, 1996). HF-HRV and RMSSD are commonly used and reliable measures of HRV. Given that the two were highly correlated (*r* = 0.89, *p* < 0.001), the current study only reports the results for HF-HRV as the main measure of HRV for this study.^2^ The values of HF-HRV were natural-log transformed to fit the assumptions of linear data for analyses.

#### 2.3.3. Perceived social support

##### 2.3.3.1. Perceived social support index

We created a perceived social support index that combined two perceived social support scales available in the dataset. These scales were both highly correlated with one another (*r* = 0.72, *p* < 0.001), indicating that they could be used together effectively as a single composite index. The perceived social support index was calculated using the 12-item Multidimensional Scale of Perceived Social Support (MSPSS; Zimet et al., 1988) and the 19-item Medical Outcomes Survey Social Support Scale (MOS; Sherbourne and Stewart, 1991). These scales were used to measure participants’ perception of the availability of their social support networks. For the MSPSS, participants responded on a seven-point Likert-type scale, ranging from 1 (*very strongly disagree*) to 7 (*very strongly agree*) on items such as “I have a special person who is a real source of comfort to me.” The MSPSS is reliable in various samples, including Latino youth (Edwards, 2004). In the current study, the reliability of the MSPSS was extremely high (α = 0.91). For the MOS, participants responded on a five-point Likert scale, ranging from 1 *(none of the time)* to 5 (*all of the time)* on items such as “Someone to give you good advice about a crisis.” The MOS is reliable in various samples such as African American and Latina American breast cancer survivors (Ashing-Giwa and Rosales, 2013). In the current study, the reliability of the MOS was extremely high (α = 0.95).

The index combining the scales was calculated by standardizing the scores within the sample across the two scales. A linear transformation to Percent of Maximum Possible (POMP) scores was used, which gave raw scores a theoretical range from 0 to 100 (Cohen et al., 1999). POMP scores were calculated by subtracting the minimum score from the raw score and then dividing by the possible scoring range. The standardized POMP scores from each perceived social support scale were then combined using an arithmetic mean. Higher scores on this index reflected higher perceived social support.

#### 2.3.4. Covariates

##### 2.3.4.1. Demographics

Self-reported sex and age were recorded, as these are sociodemographic variables known to influence both HRV (Voss et al., 2015; Koenig and Thayer, 2016) and social support (e.g., Shumaker and Hill, 1991).

### 2.4. Data analytic plan

All study analyses were conducted in SPSS (ver. 28, IBM Chicago, IL, United States). First, all variables were examined to ensure that they were normally distributed and that they were visually linearly associated with each other. Second, data points on the attachment measure falling beyond three-standard-deviations above or below the mean were identified as outliers. All attachment outliers were Winsorized by changing their value to that which was exactly three-standard-deviations above or below the mean. Analyses that refer to attachment avoidance or anxiety included the Windsorized data. Additionally, 13 extreme outliers (± 3SD) were removed on the HRV measure. Finally, descriptive statistics and bivariate associations were tested among key study variables (attachment, HRV, perceived social support) using Pearson’s *r* correlations.

To test our hypotheses, first, three simple linear regressions were conducted using SPSS to examine the linear associations of the predictor variables (attachment avoidance, attachment anxiety, and HRV) with the outcome variable (perceived social support). Next, hierarchical linear regressions were conducted via model-1 of the SPSS PROCESS macro (Hayes, 2017). This model allowed testing for two-way interactions at three different levels of the moderator (1SD below the mean, the mean, and 1SD above the mean). Attachment insecurity was entered into the model as a predictor. To assess the attachment insecurity subscales independently, one subscale was entered as a predictor while the other was entered as a statistical covariate. This approach was used to isolate the variance of the outcome variable that is predicted by each attachment style, separately (e.g., Fraley and Waller, 1998). This resulted in two models (one using attachment avoidance as the predictor and one using attachment anxiety as the predictor). For each model, the perceived social support index was entered as the outcome variable and HRV as the moderator. This established the level of HRV at which there was an association of attachment insecurity with perceived social support. Covariates for these models included age and sex to isolate the variance of the outcome variable predicted by these demographic variables. To further decompose the interactions, the predictor and moderator variable were switched. This allowed us to determine how HRV was associated with perceived social support at different levels of attachment insecurity. The above data analytic strategies were then repeated and explored solely within the Latino sample.

## 3. Results

Results from Pearson’s *r* correlations (see Table 1) revealed that, in the overall sample: older age was significantly associated with lower HRV; higher attachment avoidance was significantly associated with higher HRV; higher attachment avoidance and anxiety were significantly associated with lower perceived social support; and higher attachment avoidance was significantly associated with higher attachment anxiety. In the Latino sample and similar to the overall sample, significant associations emerged of older age with lower HRV; higher attachment avoidance was significantly associated with lower perceived social support; and higher attachment avoidance was significantly associated with higher attachment anxiety. However, and in contrast to the overall sample, no significant associations emerged of attachment avoidance with HRV, nor of attachment anxiety with perceived social support in the Latino sample.

**Table 1:** Means, standard deviations, and zero-order correlations among key study variables.

	*M*(SD)_overall_	1	2	3	4	5	*M*(SD) _Latino_
1. Age	20.45 (1.98)	–	0.01	0.19	−0.24*	0.01	20.30 (2.01)
2. Avoidance	3.02 (0.87)	−0.03	–	0.33**	0.15	−0.42**	3.10 (0.89)
3. Anxiety	3.06 (1.27)	0.02	0.42**	–	0.18	−0.07	3.00 (1.25)
4. HF-HRV	6.10 (0.91)	−0.20*	0.17*	0.07	–	0.04	6.16 (0.91)
5. Social support	78.93 (16.93)	0.00	−0.49**	−0.23**	0.01	–	78.04 (17.25)

Avoidance and anxiety indicate attachment avoidance and anxiety, respectively. Social support indicates perceived social support. Correlations for the overall sample (*N* = 145) are presented on the left side of the table while correlations for the Latino sample (*n* = 77) are presented on the right side of the table. Bolded correlations are statistically significant. *p < 0.05. **p < 0.01.

### 3.1. Overall sample (aims H1a–H3b)

In the overall sample, higher attachment avoidance (*R*^2^ = 0.20, *b* = −8.79, *p* < 0.001, 95%CI [−11.57, −6.01]; Table 2, Panel A) and higher attachment anxiety (*R^2^* = 0.06, *b* = −3.16, *p* = 0.003, 95%CI [−5.22, −1.10]; Table 2, Panel B) were associated with lower perceived social support in regression analyses, as hypothesized. However, HRV (*R*^2^ = 0.00, *b* = 0.18, *p* = 0.91, 95%CI [−2.91, 3.27]; Table 2, Panel C) was not significantly associated with perceived social support, contrary to expectations. Moreover, the associations of attachment avoidance (*R*^2^ = 0.26, *p* < 0.00, *b* = 0.47, *p* = 0.79, 95%CI [−3.06, 3.99]; Table 3) and attachment anxiety (*R*^2^ = 0.27, *p* < 0.001, *b* = −1.59, *p* = 0.13, 95%CI [−3.68, 0.49]; Table 3) with perceived social support were not moderated by HRV.

**Table 2:** Associations of attachment insecurity and HF-HRV with perceived social support.

	Overall Sample						Latino Sample					
Predictor	*b*	*SE*	*β*	*t*	*p*	95% CI	*b*	*SE*	*β*	*t*	*p*	95% CI
*Panel A*												
*R* ^2^	0.20***						0.15***					
Constant	106.27***	4.42	–	24.07	<0.001	97.54, 114.99	102.13***	6.29	–	16.25	<0.001	89.62, 114.63
Avoidance	−8.79***	1.41	−0.45	−6.25	<0.001	−11.57, −6.01	−7.52***	1.96	−0.39	−3.83	<0.001	−11.43, −3.62
*Panel B*												
*R* ^2^	0.06**						0.01					
Constant	89.28***	3.42	–	26.10	<0.001	82.52, 96.03	82.92***	4.87	–	17.03	<0.001	73.24, 92.61
Anxiety	−3.16**	1.05	−0.24	−3.03	<0.01	−5.22, −1.10	−1.34	1.52	−0.10	−0.88	0.38	−4.37, 1.69
*Panel C*												
*R^2^*	0.00						0.001					
Constant	77.83***	9.64	–	8.07	<0.001	58.77, 96.89	73.67***	13.60	–	5.42	<0.001	46.58, 100.76
HF-HRV	0.18	1.56	0.01	0.12	0.91	−2.91, 3.27	0.71	2.19	0.04	0.32	0.75	−3.64, 5.06

**p < 0.01; ***p < 0.001.

**Table 3:** Regression analyses of the association of attachment insecurity × HF-HRV with perceived social support.

	Overall sample								Latino sample							
	Attachment avoidance × HF-HRV				Attachment anxiety × HF-HRV				Attachment avoidance × HF-HRV				Attachment anxiety × HF-HRV			
Predictor	*b*	*SE*	*β*	95% CI	*b*	*SE*	*β*	95% CI	*b*	*SE*	*β*	95% CI	*b*	*SE*	*β*	95% CI
*R* ^2^	0.26***				0.27***				0.23**				0.31***			
Constant	103.23*	41.92		20.33, 186.12	62.26*	26.91		9.05, 115.46	88.10	64.55		−40.64, 216.85	1.81	37.26		−72.50, 76.12
Avoidance	−12.25	10.97	−0.63	−33.95, 9.45	–	–	–	–	−11.44	15.82	−0.59	−42.99, 20.12	–	–	–	–
Anxiety	–	–	–	–	9.13	6.56	0.69	−3.84, 22.09	–	–	–	–	28.04**	10.28	2.04	7.53, 48.54
HF-HRV	0.09	5.84	0.01	−11.45, 11.63	6.43	3.52	0.34	−0.53, 13.39	0.80	8.74	0.04	−16.64, 18.24	14.41**	4.89	0.76	4.66, 24.16
Interaction	0.47	1.78	0.18	−3.06, 3.99	−1.59	1.06	−0.80	−3.68, 0.49	0.43	2.62	0.16	−4.79, 5.65	−4.52**	1.65	−2.26	−7.81, −1.22
Age	−0.01	0.67	−0.00	−1.33, 1.30	0.11	0.64	−0.01	−1.15, 1.38	0.03	1.03	0.00	−2.04, 2.09	0.17	0.91	0.02	−1.65, 1.98
Sex	3.60	2.56	0.11	−1.46, 8.65	3.38	2.54	0.10	−1.64, 8.40	7.39*	3.69	0.22	0.04, 14.75	6.17	3.53	0.18	−0.87, 13.21
Avoidance	–	–	–	–	−9.37***	1.58	−0.48	−12.50, −6.24	–	–	–	–	−7.99***	2.08	−0.41	−12.14, −3.84
Anxiety	−0.63	1.09	−0.05	−2.78, 1.52	–	–	–	–	0.20	1.61	0.02	−3.01, 3.42	–	–	–	–
*ΔR* ^2^	0.00				0.02				0.00				0.07			

*p < 0.05; **p < 0.01; ***p < 0.001. Avoidance and anxiety indicate attachment avoidance and attachment anxiety, respectively. The rows below the interaction indicate covariates included in the models.

### 3.2. Latino sample (Aim 4)

In the Latino sample, some associations were similar to the overall sample. Specifically, higher attachment avoidance was associated with lower perceived social support (*R*^2^ = 0.15, *b* = −7.52, *p* < 0.001, 95%CI [−11.43, −3.62]; Table 2, Panel A); HRV was not associated with perceived social support (*R*^2^ = 0.001, *b* = 0.71, *p* = 0.75, 95%CI [−3.64, 5.06]; Table 2, Panel C); and the association of attachment avoidance (*R^2^* = 0.23, *p* < 0.01, *b* = 0.43, *p* = 0.87, 95%CI [−4.79, 5.65]; Table 3) with perceived social support was not moderated by HRV.

**Figure 1:**
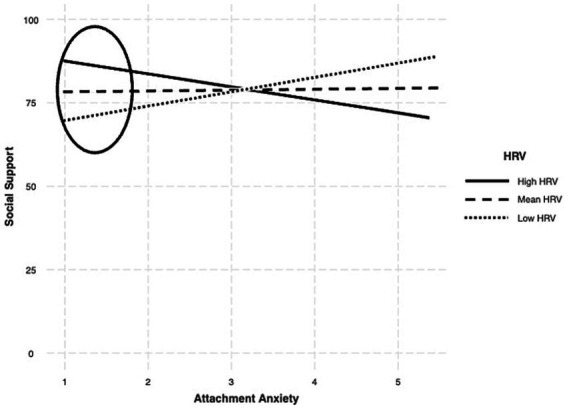
The effects of attachment anxiety on social support as a function of resting HRV among Latinos. The above figure represents the effect of attachment anxiety on perceived social support as a function of resting HRV among Latinos. Anxiety was positively associated with perceived social support at low (*b* = 4.35) but not at average (*b* = 0.23) or high (*b* = −3.88) levels of resting HRV. When switching the predictor and moderator variable, HRV was positively associated with perceived social support at low (*b* = 6.52) but not at average (*b* = 0.85) or high (*b* = −4.81) levels of attachment anxiety (indicated by the circle on the figure).

However, in the Latino sample, other associations differed from the findings in the full sample. Specifically, attachment anxiety was not associated with perceived social support (*R*^2^ = 0.01, *b* = −1.34, *p* = 0.38, 95%CI [−4.37, 1.69]; Table 2, Panel B) and the association of attachment anxiety with perceived social support was moderated by HRV in the Latino sample (*R*^2^ = 0.31, *p* < 0.001, *b* = −4.52, *p* = 0.01, 95%CI [−7.81, −1.22]; Table 3 and Figure 1). When examining the simple slopes, higher attachment anxiety was associated with higher perceived social support at low (*b* = 4.35, *p* = 0.046) but not at average (*b* = 0.23, *p* = 0.89) or high (*b* = −3.88, *p* = 0.07) levels of HRV. When switching the predictor and moderator variables, HRV was positively associated with perceived social support at low (*b* = 6.52, *p* = 0.01) but not at average (*b* = 0.85, *p* = 0.68) or high (*b* = −4.81, *p* = 0.14) levels of attachment anxiety. Overall, when attachment anxiety was lower (values between 1.0 and 2.24), it was associated with higher perceived social support but only at low levels of HRV (values between 2.82 and 4.79).

## 4. Discussion

The current study examined how attachment insecurity and HRV *together* were associated with perceived social support in a diverse young adult sample with a sizeable majority of Latinos. Three notable findings emerged. First, attachment insecurity was negatively associated with perceived social support in the overall sample, but only attachment avoidance was negatively associated with perceived social support in the Latino sample. Second, HRV was not associated with perceived social support in the overall sample nor in the Latino sample. Third, the interaction of attachment insecurity and HRV with perceived social support was not significant in the overall sample; in the Latino sample, however, the interaction of attachment anxiety and HRV with perceived support was statistically significant. These findings are a novel contribution to research on the association of attachment and HRV with perceived social support that highlight the importance of considering psychological processes, physiological processes, and socio-cultural context to better understand social support.

The observed associations of higher avoidance and anxiety with lower perceived social support in the overall sample are consistent with the literature (Mallinckrodt and Wei, 2005; Mikulincer and Shaver, 2009; Stanton and Campbell, 2014). However, in the Latino sample, only higher avoidance, not anxiety, was associated with lower perceived social support. That pattern is consistent with other research indicating that attachment avoidance is particularly incongruent with Latino culture (Friedman et al., 2010; Campos et al., 2016). In this cultural context, wherein interdependence is normative and valued, individuals higher in attachment avoidance may find their social worlds to be particularly at odds with their internal working models. Higher attachment avoidance is manifested by a preference for maintaining distance and self-reliance that minimizes closeness and interdependence (Mikulincer and Shaver, 2010), but this may be more difficult to do when one’s cultural context emphasizes interdependence and socializes practices that foster closeness. A Latino person who is higher in avoidance may feel overwhelmed by their social environment. In contrast, the association of attachment anxiety with perceived social support was not observed in the Latino sample. This pattern is also consistent with research that suggests that being higher in attachment anxiety is less problematic in Latino culture (Campos et al., 2016); in this context, people higher in attachment anxiety are likely to engage with a social environment that emphasizes interdependence and readily available social support. Null findings warrant care against the possibility of overinterpretation, but the overall patterns observed in the Latino sample in this study suggest that there is a great deal to still be learned about attachment processes and social support by focusing on this cultural context. These findings also suggest that there is an overall need to better understand the role of sociocultural variation in the links of attachment, HRV, and perceived social support.

Surprisingly, HRV was not associated with perceived social support in the overall sample or the Latino sample. This finding is contrary to expectations. A recent review of this literature by Goodyke et al. (2022) indicates that higher HRV is associated with higher perceived social support. In that same review, Goodyke et al. (2022) suggest that there should be an increased call for diversity of health status in samples. The findings of this study are additional evidence of the need for a focus on diverse samples. Most of the work examining the link between HRV and perceived social support has been conducted with European or European American samples (e.g., Schwerdtfeger and Schlagert, 2011), a distinct sociocultural context that emphasizes cultural independence and wherein maximizing the potential benefits of social support might pose distinct challenges (e.g., Bolger and Amarel, 2007; Campos et al., 2018). Our diverse overall sample was majority Latino, and one possibility is that there may be distinct patterns in the association of HRV with perceived social support in Latinos. Another possibility is that HRV, a physiological index of self-regulation, may not relate to perceived social support in all contexts. It may be that HRV relates to perceived social support only when specific psychological experiences are also at play (e.g., under stressful conditions). More studies that examine HRV and perceived social support in samples that are ethnically diverse, health status diverse, and diverse in terms of context-specific psychological factors are needed to better understand how this important link may be differentially associated in various groups.

Interestingly, in Latinos with lower self-regulatory abilities, as indexed by lower levels of HRV, there was a significant association of higher attachment anxiety with higher perceived social support. This association was only significantly different from zero at lower levels of attachment anxiety. When attachment anxiety and HRV were lower, perceived social support was significantly higher than average, whereas when attachment anxiety was at average levels or higher, HRV was not significantly associated with perceived social support. This finding suggests that Latino participants who had a lower ability to self-regulate, as indexed by lower HRV, perceived higher levels of social support when they endorsed *some* attachment anxiety rather than absolutely none. HRV has been previously conceptualized as an endophenotype, highlighting the trait-like, yet modifiable, nature of this biomarker over the lifespan (Beauchaine and Thayer, 2015). Given that Latinos generally report higher levels of socially supportive networks (Campos and Kim, 2017), those with lower HRV who grow up in this cultural context may experience some degree of stress-buffering. The generally supportive and accessible nature of Latino culture may allow individuals who endorse higher levels of attachment insecurity to still perceive high levels of social support even in the presence of lower HRV. We should note, however, that this pattern was only observed when attachment insecurity was below average suggesting that the cultural context does not buffer individuals who are *highly* insecure. We speculate that at higher levels of attachment anxiety, when a person’s needs are most likely to exceed their resources, similar levels of social support may be perceived, irrespective of HRV. More work needs to be done that examines how the interaction between attachment insecurity and HRV may be associated with perceived social support in larger samples of Latinos and other minoritized ethnic/cultural groups, but the intriguing results of the present study suggest that cultural context is important.

We are mindful that there are also additional cultural contexts in which varying patterns are likely to be observed. For example, individuality and the pursuit of personal goals tend to be valued among European Americans (Markus and Kitayama, 1991). It is possible that the patterns of attachment, HRV, and perceptions of social support may function differently in this cultural context. Similarly, East Asian cultures, like Latino cultures, value interdependence, but the two endorse different forms of interdependence (Campos and Kim, 2017). There is also intriguing empirical indication that avoidant attachment is prevalent and may meaningfully differ in East Asian culture from patterns commonly observed in samples of European heritage (Wei et al., 2004). A full exploration of these possibilities could not be conducted in this study due to the smaller size, and thus, limited statistical power, of the non-Latino sample (i.e., participants of European and East Asian heritage). However, we hope that the innovative associations studied in the present work inspire researchers to study the links of attachment, HRV, and perceptions of social support in other ethnic groups in future research.

### 4.1. Strengths, limitations, and future directions

The findings of this study should be considered with a few limitations in mind. Although we had a diverse sample, all were young adults. This may limit the generalizability of these findings; future research should consider a more age- and generation-diverse sample. Additionally, our study’s sample size was relatively small. This may have limited statistical power in our study. Nevertheless, we had an ethnically diverse sample comprised of individuals of East Asian, European, and Latino heritage. As culture/ethnicity play a large role in psychological and relational processes (Campos and Kim, 2017), this diverse dataset allowed us to better understand nuances in social support processes. A clear implication of this work is that researchers must recruit people of diverse backgrounds, and ethnically minoritized backgrounds specifically, into their studies. This may be a formidable goal, and it may involve encouraging the field to reward scientists who forego more convenient samples to take on the labor-intensive challenge of recruiting diverse samples. Doing so, however, stands to substantially advance current knowledge and move the field closer to a comprehensive science of human psychology, including the social support processes studied here.

## 5. Conclusion

Overall, this study contributes to a better understanding of psychological and physiological indicators of socio-emotional self-regulation that are both, in tandem, associated with perceived social support. As such, this study is among the first to consider how attachment insecurity and HRV together are associated with perceived social support, and the first to do so in a diverse sample that was majority Latino, a cultural context that is particularly incongruent with attachment avoidance and in which affordances may buffer the challenges associated with attachment anxiety. We hope these findings inspire a much-needed focus on recruiting more diverse samples into studies and to consider how psychological and physiological factors together relate to social support, a psychological process robustly linked with downstream beneficial health outcomes that are universally important.

## Data availability statement

The datasets presented in this article are not readily available because the sample is unique, and participant re-identification is possible. The raw data supporting the conclusions of this article can be made available by the authors upon reasonable request. Requests to access the datasets should be directed to BC, bcampos@uci.edu.

## Ethics statement

The studies involving humans were approved by the University of California, Irvine Institutional Review Board. The studies were conducted in accordance with the local legislation and institutional requirements. The participants provided their written informed consent to participate in this study.

## Author contributions

BC and IY contributed to conception, design, and data collection of the study. VP organized the database and performed the statistical analysis. VP and NF wrote the first draft of the manuscript. VP, NF, and DW contributed to the conception of the manuscript. All authors contributed to manuscript writing, revision, and approved the submitted version.

## Footnotes

^1^Hereafter referred to as “HRV” unless specified otherwise.

^2^Results did not change when analyses were conducted using RMSSD.
